# Usefulness of health checkup‐based indices in identifying metabolic dysfunction‐associated steatotic liver disease

**DOI:** 10.1002/jgh3.13110

**Published:** 2024-06-17

**Authors:** Takao Miwa, Satoko Tajirika, Nanako Imamura, Miho Adachi, Ryo Horita, Tatsunori Hanai, Cheng Han Ng, Mohammad Shadab Siddiqui, Taku Fukao, Masahito Shimizu, Mayumi Yamamoto

**Affiliations:** ^1^ Health Administration Center Gifu University Gifu Japan; ^2^ Department of Gastroenterology/Internal Medicine, Graduate School of Medicine Gifu University Gifu Japan; ^3^ Division of Gastroenterology and Hepatology, Department of Medicine National University Hospital Singapore Singapore; ^4^ Division of Gastroenterology, Hepatology and Nutrition, Department of Internal Medicine Virginia Commonwealth University Richmond Virginia USA; ^5^ United Graduate School of Drug Discovery and Medical Information Sciences Gifu University Gifu Japan

**Keywords:** biomarker, lean, non‐alcoholic fatty liver disease, non‐alcoholic steatohepatitis, screening

## Abstract

**Aims:**

The application of indices in the context of metabolic dysfunction‐associated steatotic liver disease (MASLD) remains unexplored. We aimed to validate the ability of alanine aminotransferase (ALT), fatty liver index (FLI), and hepatic steatosis index (HSI) to identify MASLD during health checkups.

**Methods:**

We recruited 627 participants and utilized their health checkup data and ultrasound to assess the potential of using ALT, FLI, and HSI as indices for MASLD; this was indicated by the area under the curve (AUC) and restricted cubic spline (RCS) model. The optimal, rule‐out (sensitivity ≥90%), and rule‐in (specificity ≥90%) cutoff values of each index for identifying MASLD were reported.

**Results:**

Among participants with a median age of 46 years, the prevalence of MASLD was 28% in total (38% in males and 18% in females). RCS models confirmed a linear association between indices and MASLD. ROC analyses indicated that the AUC of ALT in identifying MASLD was 0.79 for the total cohort, 0.81 for males, and 0.69 for females. The optimal, rule‐out, and rule‐in cutoff values for ALT were 21, 13, and 29, respectively. Similarly, the AUC of FLI/HSI in identifying MASLD was 0.90/0.88 for the total cohort, 0.86/0.85 for males, and 0.93/0.90 for females. Considering the reference cutoff values, distinct cutoff values were observed between the sexes for FLI, while HSI had similar cutoff values.

**Conclusion:**

This study demonstrated that ALT > 30 IU/L is a reasonable cutoff value to rule‐in MASLD. ALT, FLI, and HSI are reliable indices for identifying MASLD during health checkups.

## Graphical abstract

The “Nara declaration” recommends further investigations to evaluate chronic liver disease in individuals with serum alanine aminotransferase (ALT) levels > 30 IU/L. Our study evaluated the validity of the Nara declaration suggesting the use of serum ALT > 30 IU/L to identify metabolic dysfunction‐associated steatotic liver disease (MASLD) and further provided valuable evidence to validate the ability of fatty liver index and hepatic steatosis index to act as indices in identifying MASLD during health checkups.

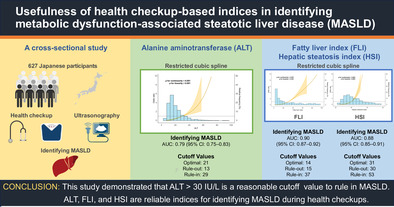

## Introduction

Due to the rising rates of obesity, the prevalence of non‐alcoholic fatty liver disease (NAFLD) and non‐alcoholic steatohepatitis is rapidly increasing along with type 2 diabetes, hypertension, and dyslipidemia, resulting in an increased burden of liver cirrhosis, hepatocellular carcinoma, and mortality attributed to these conditions.^1^, ^2^, ^3^ With accumulating evidence of metabolic risk factors, NAFLD was recently renamed to metabolic dysfunction‐associated steatotic liver disease (MASLD) to avoid the reliance on exclusionary confounder terms and the use of potentially stigmatizing language.^4^, ^5^ While NAFLD is defined by the exclusion of other liver diseases, MASLD is a diagnostic criterion based on the high‐risk factors in a patient's profile.^4^, ^5^, ^6^, ^7^, ^8^ Therefore, MASLD is expected to identify individuals at a high risk of liver‐related outcomes in the future.

The prevalence of MASLD is reported to be 26–30% in Asian health checkup population.^9^, ^10^, ^11^ As lifestyle interventions such as dietary and exercise therapy are the cornerstone of all clinical therapy for MASLD and NAFLD,^6^, ^7^, ^8^ health checkups can be an important opportunity for early detection of MASLD and implementing behavioral changes to positively affect the natural history of the disease and reduce long‐term complications. However, performing ultrasound on all individuals during health checkups is impractical because of the cost and time involved. Recently, the Japan Society of Hepatology announced the “Nara declaration,” which recommends further investigations to evaluate chronic liver disease in individuals with serum alanine aminotransferase (ALT) levels >30 IU/L to identify the etiology of chronic liver disease.^12^ However, there is an urgent need for validation studies to explore the usefulness of serum ALT levels >30 IU/L in the context of MASLD. Furthermore, the fatty liver index (FLI) and hepatic steatosis index (HSI) were originally developed as noninvasive tests to identify NAFLD.^13^, ^14^ These tests have been used as alternative indicators for NAFLD in large‐scale epidemiological studies and health screenings.^15^, ^16^, ^17^, ^18^ Recent data suggest that almost all patients with NAFLD also have MASLD, and that the clinical impact on cardiovascular risks, extrahepatic comorbidities, and mortality is similar between the two conditions.^19^, ^20^, ^21^, ^22^, ^23^, ^24^, ^25^, ^26^ Therefore, these tests may also be used to identify MASLD in the same manner. However, with the renaming of NAFLD to MASLD, it is not clear whether these indices remain useful considering the new definition of MASLD.

The primary aim of this study was to evaluate the discriminative ability of ALT to identify MASLD during health checkups. The secondary aim was to validate the ability of FLI and HSI to identify MASLD during health checkups.

## Methods

### Study design and participants

This cross‐sectional study assessed 831 university staff and faculty members at Gifu University (general and national university in Gifu, Japan) for eligibility. Finally, it included 627 participants based on the inclusion and exclusion criteria (Fig. S1). The inclusion criteria were university staff and faculty members who underwent an occupational annual health checkup that was mandatory based on the Occupational Health and Safety Act in June 2023 and those with written informed consent. The exclusion criteria were self‐identified non‐Japanese; neuromuscular diseases; alcohol intake greater than that allowed for MASLD/NAFLD (male >30 g/day and female >20 g/day)^4^, ^5^; viral hepatitis, autoimmune hepatitis, and primary biliary cholangitis; severe comorbidities; and those with missing data. The study objectives were thoroughly explained and written informed consent was obtained from all participants. The study protocol was reviewed and approved by the institutional review board of the Graduate School of Medicine, Gifu University (approval no. 2022‐237). The study conformed to the Declaration of Helsinki and was reported in accordance with the Strengthening the Reporting of Observational Studies in Epidemiology guidelines.

### Clinical and laboratory evaluations

Health checkup data were collected to evaluate the clinical and laboratory data. The alcohol intake allowed for MASLD/NAFLD (male ≤30 g/day and female ≤20 g/day) was considered mild alcohol intake based on the consensus statement.^4^, ^5^ Weight, height, and waist circumference were assessed at the time of health checkup. Laboratory evaluation included aspartate aminotransferase (AST), ALT, gamma‐glutamyl transferase (GGT), triglycerides (TG), high‐density lipoprotein (HDL) cholesterol, low‐density lipoprotein cholesterol, hemoglobin A1c (HbA1c), fasting or occasional glucose levels, and platelet count.

### Disease definitions

Hepatic steatosis was assessed by hepatologists and medical technologists, blinded to the laboratory data, using liver ultrasound (LOGIQ P10; GE Healthcare Japan, Tokyo, Japan). The diagnosis of MASLD was made based on the consensus statement.^4^, ^5^ Participants with hepatic steatosis and any of the following cardiometabolic criteria were diagnosed with MASLD: overweight, impaired glucose tolerance, hypertension, hypertriglyceridemia, and low HDL cholesterol. Overweight was defined as body mass index (BMI) ≥ 23 kg/m^2^. Elevated waist circumference was evaluated as >94 cm in males and >80 cm in females. Impaired glucose tolerance was defined as fasting serum glucose level ≥100 mg/dL, occasional glucose level ≥140 mg/dL, HbA1c ≥5.7%, presence of type 2 diabetes, or taking any antidiabetic treatment. Hypertension was defined as blood pressure ≥130/85 mmHg or the use of antihypertensive medication. Hypertriglyceridemia was defined as plasma TG ≥ 150 mg/dL or the use of antihypertriglyceridemic medication. Low HDL cholesterol was defined as plasma HDL cholesterol <40 mg/dL in males and <50 mg/dL in females or taking any antidyslipidemic treatment.^4^, ^5^ The diagnosis of NAFLD was based on the guidelines for NAFLD.^6^, ^7^, ^8^ Participants with hepatic steatosis and no other liver diseases were diagnosed with NAFLD. When participants showed indications of liver disease, referral letters were written to medical institutions for the exclusion of other liver diseases.

### Assessment of health checkup‐based indices

Using the data obtained from health checkups, FLI was calculated using the following formula: FLI = (e^{0.953*loge [TG]+0.139*BMI+0.718*loge [GGT]+0.053*waist circumference−15.745}^)/(1 + e^{0.953*loge [TG]+0.139*BMI+0.718*loge [GGT]+0.053*waist circumference−15.745}^) * 100.^13^ In the original report, the rule‐out and rule‐in cutoff values of FLI were reported as 30 and 60, respectively. Similarly, HSI was evaluated using the following formula: HSI = 8 * (ALT/AST) + BMI (+2 if female, +2 if type 2 diabetes).^14^ In the original report, the rule‐out and rule‐in cutoff values of HSI were reported as 30 and 36, respectively. In addition, the fibrosis‐4 (FIB‐4) index was evaluated using the following formula: (age * AST)/(platelet [10^9^/L] * ALT^1/2^).^27^

### Statistical analyses

Quantitative and qualitative variables were reported as median (interquartile range) and number of patients (percentage), respectively. Participants with and without MASLD were compared using the chi‐square test or Mann–Whitney *U* test. The discriminative ability of ALT, FLI, and HSI was evaluated using the receiver operating curve (ROC) analysis and shown as the area under the curve (AUC). The Youden's index was used to determine the optimal cutoff value for identifying MASLD, and the sensitivity, specificity, positive predictive value (PPV), and negative predictive value (NPV) were demonstrated in each cutoff value. The discriminative ability when using the 90% sensitivity (rule‐out value), 90% specificity (rule‐in value), and the reference values of ALT, FLI, and HSI were also reported.^12^, ^13^, ^14^ A restricted cubic spline (RCS) model with three knots adjusted by age and sex was applied to demonstrate the association between the values of ALT, FLI, and HSI and the odds ratio of MASLD. Given the reference data,^12^, ^13^, ^14^ the reference point was adjusted to 30 and the bin width of the histogram was 5 in ALT and FLI and 2 in HSI. Nonlinearity was evaluated using the likelihood ratio test comparing a model including both linear and cubic spline terms and a model containing only linear terms.^28^ All analyses were two‐sided and *p* < 0.05 was set as the threshold for statistical significance. All analyses were performed using JMP (version 17.0.0, SAS Institute Inc., Cary, NC, USA) and R (version 4.3.1 software, The R Foundation for Statistical Computing, Vienna, Austria).

### Sample size calculation

To conduct a multivariate analysis for the RCS model including age, sex, and ALT, FLI, or HSI, a minimum of 30 participants with MASLD were necessary in our study. Given the estimated 20% prevalence of MASLD among the Japanese health checkup cohort, we calculated that a minimum sample size of 150 participants is required to evaluate the association between MASLD and ALT, FLI, or HSI in our study.

## Results

### Characteristics of participants

The characteristics of the 627 participants are shown in Table 1. The median age of the participants was 46 years, 52% were male, and 44% were mild alcohol drinkers. The median waist circumference and BMI were 78 cm and 23 kg/m^2^, respectively. The prevalence of overweight, elevated waist circumference, impaired glucose tolerance, hypertension, hypertriglyceridemia, and low HDL cholesterol was 44, 20%, 20%, 28%, 31%, and 17%, respectively. Regarding health checkup‐based indices, the median ALT, FLI, and HSI were 17, 12, and 30, respectively.

**Table 1:** Characteristics of participants categorized based on MASLD

Characteristics	Participants	No MASLD	MASLD	*p‐*value
	(*n* = 627)	(*n* = 450)	(*n* = 177)	
Demographics				
Age (years)	46 (36–53)	44 (34–53)	49 (40–54)	0.003
Male sex	328 (52)	204 (45)	124 (70)	<0.001
Mild alcohol intake, *n* (%)	275 (44)	193 (43)	82 (46)	0.435
Physical examination				
Waist circumference (cm)	78 (71–85)	75 (68–81)	88 (82–96)	<0.001
Body mass index (kg/m^2^)	23 (20–25)	21 (19–23)	26 (24–30)	<0.001
Cardiometabolic criteria				
Overweight, *n* (%)	278 (44)	124 (28)	154 (87)	<0.001
Elevated waist circumference (%)	128 (20)	44 (10)	84 (47)	<0.001
Impaired glucose tolerance, *n* (%)	123 (20)	58 (13)	65 (37)	<0.001
Hypertension, *n* (%)	173 (28)	68 (15)	105 (59)	<0.001
Hypertriglyceridemia, *n* (%)	192 (31)	81 (18)	111 (63)	<0.001
Low HDL cholesterol, *n* (%)	108 (17)	41 (9)	67 (38)	<0.001
Laboratory test				
AST (IU/L)	18 (15–22)	17 (15–20)	21 (18–27)	<0.001
ALT (IU/L)	17 (13–26)	15 (12–20)	27 (19–42)	<0.001
Male	22 (16–33)	18 (14–25)	34 (22–49)	<0.001
Female	14 (11–19)	14 (11–17)	17 (13–27)	<0.001
GGT (IU/L)	30 (14–31)	17 (13–24)	32 (22–49)	<0.001
Creatinine (mg/dL)	0.77 (0.67–0.93)	0.74 (0.65–0.91)	0.85 (0.73–0.98)	<0.001
Triglycerides (mg/dL)	102 (70–157)	87 (61–125)	171 (113–241)	<0.001
HDL cholesterol (mg/dL)	60 (49–71)	64 (56–76)	47 (43–57)	<0.001
LDL cholesterol (mg/dL)	113 (96–132)	109 (92–127)	125 (108–148)	<0.001
HbA1c (%)	5.4 (5.2–5.6)	5.3 (5.2–5.5)	5.5 (5.3–5.8)	<0.001
Platelet (×10^3^/μL)	260 (228–299)	253 (221–288)	274 (244–324)	<0.001
Noninvasive test				
Fatty liver index	12 (5–39)	7 (3–17)	54 (27–77)	<0.001
Male	28 (9–57)	15 (6–32)	59 (39–79)	<0.001
Female	5 (2–13)	5 (2–8)	30 (15–69)	<0.001
Hepatic steatosis index	30 (27–35)	28 (26–31)	37 (33–43)	<0.001
Male	33 (29–38)	31 (28–34)	39 (34–44)	<0.001
Female	28 (26–31)	27 (25–29)	34 (31–39)	<0.001
FIB‐4 index	0.73 (0.54–0.98)	0.74 (0.54–1.04)	0.69 (0.53–0.87)	0.027
Male	0.76 (0.56–1.00)	0.80 (0.58–1.06)	0.70 (0.53–0.90)	0.010
Female	0.67 (0.51–0.94)	0.69 (0.51–0.99)	0.67 (0.53–0.83)	0.272

Values are presented as number (percentage) or median (interquartile range).

ALT, alanine amino transferase; AST, aspartate aminotransferase; FIB‐4, fibrosis‐4; GGT, gamma‐glutamyl transferase; HbA1c, hemoglobin A1c; HDL, high‐density lipoprotein; LDL, low‐density lipoprotein; MASLD, metabolic dysfunction‐associated steatotic liver disease.

### Prevalence and characteristics of MASLD

The prevalence of MASLD was 28% (*n* = 177) in total cohort, 38% in males (*n* = 124), and 18% in females (*n* = 53). Overlap of MASLD and NAFLD was observed in 28% (*n* = 177) of the participants, while 2% (*n* = 11) had only NAFLD (Fig. S2).

The comparison between participants with and without MASLD is shown in Table 1. Participants diagnosed with MASLD were characterized by older age, a higher proportion of males, elevated levels of serum AST, ALT, GGT, creatinine, TG, LDL cholesterol, HbA1c, and platelet count, along with lower levels of HDL cholesterol compared with those without MASLD. In particular, serum ALT levels were significantly higher in those with MASLD than those without MASLD (27 vs. 15 IU/L; *p* < 0.001). Furthermore, the FLI (54 vs. 7; *p* < 0.001) and HSI (37 vs. 28; *p* < 0.001) were significantly higher in those with MASLD than those without MASLD (Table 1).

### Impact of ALT on the odds ratio of MASLD

**Figure 1:**
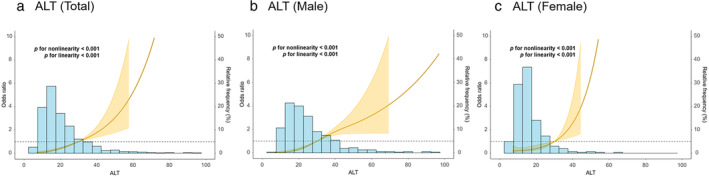
Restricted cubic spline models show the ability of alanine aminotransferase (ALT) to identify metabolic dysfunction‐associated steatotic liver disease (MASLD) in (a) total cohort, (b) males, and (c) females.

RCS models were applied to assess the influence of ALT on the odds ratio of MASLD. In the total cohort, the RCS model demonstrated that the odds ratio of MASLD increases with increasing ALT values (Fig. 1a). Similarly, the odds ratio of MASLD increases with rising serum ALT levels in males (Fig. 1b) and females (Fig. 1c).

### Discriminative ability of ALT to identify MASLD

In the total cohort, ROC analyses indicated that the AUC for ALT in identifying MASLD was 0.79. In sex‐stratified analysis, the AUC for ALT in identifying MASLD was 0.81 for males and 0.69 for females.

The cutoff values and the discriminative ability of ALT to identify MASLD are presented in Table 2. In the total cohort, the optimal, rule‐out, and rule‐in cutoff values of ALT for identifying MASLD were 21, 13, and 29, respectively. Sex‐stratified analysis showed that the optimal, rule‐out, and rule‐in cutoff values of ALT for identifying MASLD were 21, 18, and 33 for males and 23, 11, and 37 for females, respectively.

**Table 2:** Discriminative ability of ALT to identify MASLD

	ALT				
	Cutoff value	Sensitivity	Specificity	PPV	NPV
Total cohort					
Optimal	21	71	76	53	87
Rule‐out	13	93	28	34	91
Rule‐in	29	47	92	72	81
Predetermined	30	46	91	68	81
Male					
Optimal	21	84	63	58	87
Rule‐out	18	90	45	50	88
Rule‐in	33	53	92	81	76
Predetermined	30	58	87	73	77
Female					
Optimal	23	40	91	48	88
Rule‐out	11	93	23	21	93
Rule‐in	37	9	99	71	84
Predetermined	30	19	97	56	85

Values are presented as percentage. The discriminative ability of ALT was assessed at the optimal cutoff, rule‐out cut off (sensitivity ≥90%), and rule‐in cutoff (specificity ≥90%), and predetermined cutoff of 30.

ALT, alanine amino transferase; MASLD, metabolic dysfunction‐associated steatotic liver disease; NPV, negative predictive value; PPV, positive predictive value.

### Comparison between ALT and FIB‐4 index

**Figure 2:**
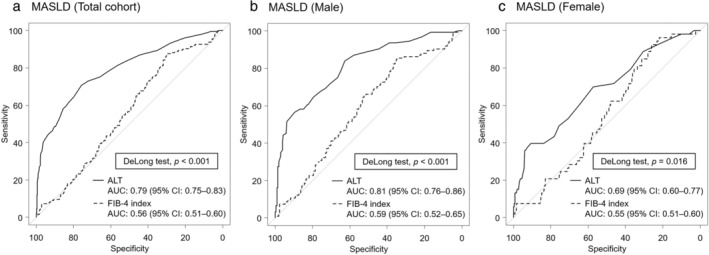
Discriminative ability of alanine aminotransferase (ALT) and FIB‐4 index in identifying metabolic dysfunction‐associated steatotic liver disease (MASLD) in (a) total cohort, (b) males, and (c) females. AUC, area under the curve; CI, confidence interval; FIB‐4, fibrosis‐4; HSI, hepatic steatosis index.

In the total cohort, ROC analyses indicated that the AUC for FIB‐4 index in identifying MASLD was 0.56. In sex‐stratified analysis, the AUC for ALT in identifying MASLD was 0.59 for males and 0.55 for females. The RCS models indicated inverse association between the FIB‐4 index and MASLD (Fig. S3). The DoLong test showed that ALT was better than FIB‐4 index in identifying MASLD in total cohort (Fig. 2a), males (Fig. 2b), and females (Fig. 2c).

### Impact of FLI and HSI on the odds ratio of MASLD

**Figure 3:**
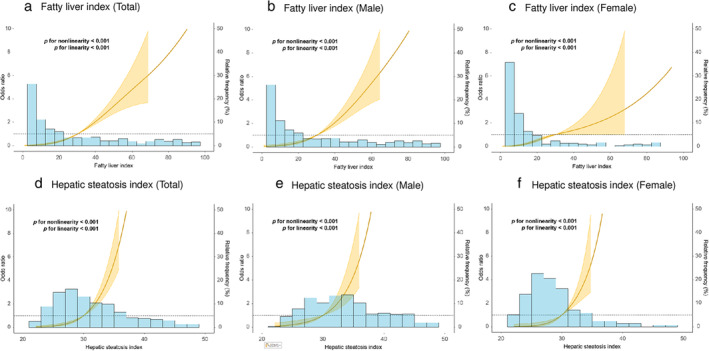
Restricted cubic spline models show the ability of fatty liver index (FLI) to identify metabolic dysfunction‐associated steatotic liver disease (MASLD) in (a) total cohort, (b) males, and (c) females, and the ability of hepatic steatosis index (HSI) to identify MASLD in (d) total cohort, (e) males, and (f) females.

RCS models were assessed to evaluate the influence of FLI and HSI on the odds ratio of MASLD. Regarding FLI, the RCS model demonstrated that the odds ratio of MASLD increases with increasing FLI values in the total cohort (Fig. 3a), in males (Fig. 3b), and in females (Fig. 3c). Similarly, the odds ratio of MASLD also increases with rising HSI values in the total cohort (Fig. 3d), in males (Fig. 3e), and females (Fig. 3f).

### Discriminative ability of FLI and HSI to identify MASLD

**Figure 4:**
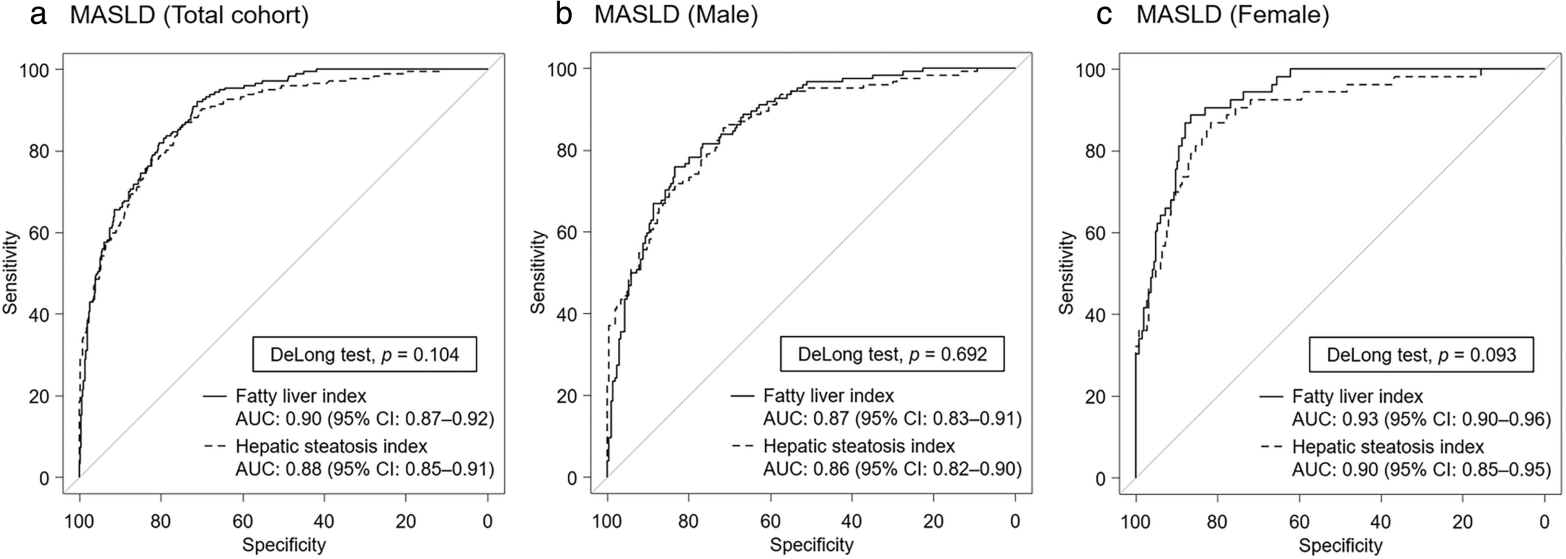
Discriminative ability of fatty liver index (FLI) and hepatic steatosis index (HSI) in identifying metabolic dysfunction‐associated steatotic liver disease (MASLD) in (a) total cohort, (b) males, and (c) females. AUC, area under the curve; CI, confidence interval.

In the total cohort, ROC analyses indicated that the AUC for FLI and HSI in identifying MASLD was 0.90 and 0.88, respectively (Fig. 4a). In the sex‐stratified analysis, the AUC for FLI and HSI in identifying MASLD was 0.87 and 0.86 for males (Fig. 4b) and 0.93 and 0.90 for females (Fig. 4c), respectively. The DeLong test showed no significant difference between FLI and HSI.

The cutoff values and discriminative ability of FLI and HSI in identifying MASLD are presented in Table 3. Regarding FLI, the optimal, rule‐out, and rule‐in cutoff values of FLI for identifying MASLD were 14, 15, and 37, respectively. Sex‐stratified analysis showed that the optimal, rule‐out, and rule‐in cutoff values of FLI for identifying MASLD were 39, 21, and 53 for males and 12, 10, and 15 for females, respectively. As for HSI, the optimal, rule‐out, and rule‐in cutoff values for identifying MASLD were 31, 30, and 35, respectively. Sex‐stratified analysis showed that the optimal, rule‐out, and rule‐in cutoff values of HSI for identifying MASLD were 33, 32, and 38 for males and 30, 29, and 32 for females, respectively.

**Table 3:** Discriminative ability of fatty liver index and hepatic steatosis index to identify MASLD

	Fatty liver index					Hepatic steatosis index				
	Cutoff value	Sensitivity	Specificity	PPV	NPV	Cutoff value	Sensitivity	Specificity	PPV	NPV
Total cohort										
Optimal	14	91	71	56	95	31	88	72	55	94
Rule‐out	15	91	72	56	95	30	93	64	50	96
Rule‐in	37	66	90	72	87	35	62	90	71	86
Predetermined	30	73	85	66	89	30	93	64	50	96
Predetermined	60	43	97	86	81	36	57	94	78	85
Male										
Optimal	39	76	83	73	85	33	86	69	63	89
Rule‐out	21	91	61	59	92	32	91	60	58	92
Rule‐in	53	59	90	79	78	38	55	92	81	77
Predetermined	30	83	72	64	88	30	95	43	50	94
Predetermined	60	50	94	84	76	36	65	87	76	81
Female										
Optimal	12	87	87	59	97	30	86	81	50	97
Rule‐out	10	91	81	51	98	29	91	73	42	97
Rule‐in	15	77	90	62	95	32	59	92	62	91
Predetermined	30	49	96	74	90	30	86	81	50	97
Predetermined	60	26	100	100	86	36	36	99	86	88

Values are presented as percentage. The discriminative ability of fatty liver index was assessed at the optimal cutoff, rule‐out cut off (sensitivity ≥90%), rule‐in cutoff (specificity ≥90%), and predetermined cutoff of 30 and 60. The discriminative ability of hepatic steatosis index was assessed at the optimal cutoff, rule‐out cut off (sensitivity ≥90%), rule‐in cutoff (specificity ≥90%), and predetermined cutoff of 30 and 36.

MASLD, metabolic dysfunction‐associated steatotic liver disease; NPV, negative predictive value; PPV, positive predictive value.

### Discriminative ability of indices to identify obese and nonobese MASLD

In obese (BMI ≥ 25 mg/m^2^) participants, ROC analyses indicated that the AUC for ALT, FLI, and HSI in identifying obese MASLD was 0.74, 0.77, and 0.78, respectively. In the sex‐stratified analysis, the AUC for ALT, FLI, and HSI in identifying obese MASLD was 0.76, 0.75, and 0.79 for males and 0.71, 0.81, and 0.76 for females, respectively. The cutoff values and discriminative ability of ALT, FLI, and HSI in identifying obese MASLD are presented in Tables S1 and S2.

In nonobese (BMI < 25 mg/m^2^) participants, ROC analyses indicated that the AUC for ALT, FLI, and HSI in identifying nonobese MASLD was 0.73, 0.86 and 0.83, respectively. In the sex‐stratified analysis, the AUC for ALT, FLI, and HSI in identifying nonobese MASLD was 0.77, 0.82, and 0.79 for males and 0.62, 0.91, and 0.85 for females, respectively. The cutoff values and discriminative ability of ALT, FLI, and HSI in identifying nonobese MASLD are presented in Tables S3 and S4.

## Discussion

To the best of our knowledge, this is the first study to investigate the ability of ALT, FLI, and HSI to identify MASLD in a large‐scale population health dataset. Few studies have been performed on the association between MASLD and health checkup‐based indices to identify MASLD. Despite the recent name change and diagnostic criteria, the primary finding of this study supports the use of ALT as a simple biomarker to identify MASLD in health checkup settings. Furthermore, FLI and HSI were found to be accurate in indicating the presence of MASLD. These findings expand our understanding and provide a potential strategy to identify MASLD in health checkup settings.

Given the recent change in nomenclature from NAFLD to MASLD, there remains a lack of understanding regarding the clinical characteristics of MASLD and the distinctions between MASLD and NAFLD. In our study, we observed that only 2% of the participants had NAFLD alone and the majority of participants had overlapping MASLD and NAFLD. This result significantly reveals that the population with MASLD does not differ greatly from that with NAFLD. Therefore, our results imply that the accumulated evidence related to NAFLD can be similarly applied to MASLD in the Japanese population.

Our study evaluated the usefulness of serum ALT levels in identifying MASLD during health checkups. The RCS models illustrated that the odds ratio of MASLD increases with rising ALT in both sexes. The optimal cutoff values of ALT were lower than the general reference value of ALT. However, applying the optimal cutoff values to health checkup settings may not be appropriate because of the large number of false positives and cost‐effectiveness. Therefore, we assessed the rule‐in cutoff value, which has high specificity and is generally used to establish reliable indices.^13^, ^14^ Notably, in our study, the cutoff value of ALT for identifying MASLD was close to ALT 30 IU/L. Therefore, our results support the “Nara declaration” of the Japan Society of Hepatology, which recommended further evaluation in individuals with ALT > 30 IU/L. The results of our study are also supported by the guidelines for the evaluation of abnormal liver chemistries previously clarified that normal ALT level ranges from 29 to 33 IU/L in males and 19 to 25 IU/L in females.^29^ Furthermore, recent evidence suggests that ALT > 30 IU/L is also effective in identifying significant fibrosis among individuals aged ≥65 years.^30^ Therefore, our results imply that simple measurement of ALT is a useful method to identify MASLD in health checkup settings and is in accordance with previous evidence regarding NAFLD.

Identifying significant fibrosis is essential to improve the outcome of those with MASLD and the FIB‐4 index plays an important role as a surrogate marker for significant fibrosis in health checkup population.^31^, ^32^, ^33^ However, there is a contradiction that the mean (or median) FIB‐4 index was lower in those with MASLD than those without in previous studies.^9^, ^32^ This seems reasonable because generally AST is higher than ALT in healthy people and the AST/ALT ratio decreases when they develop MASLD.^34^ Furthermore, the AST/ALT ratio elevates as a result of progression to cirrhosis when hepatocyte ballooning and steatosis may disappear.^34^, ^35^ Because the FIB‐4 index correlates with ALT/ALT ratio, it should be emphasized that the FIB‐4 index may not be suitable for identifying MASLD in early stage. Given that health checkups aim to detect diseases early and encourage lifestyle intervention, it is essential to identify MASLD at an early stage.^36^, ^37^, ^38^ Our results indicate that simple measurement of ALT is better than the FIB‐4 index in terms of identifying MASLD in an early stage in health checkup setting.

Our study also revealed that both FLI and HSI were useful in identifying MASLD during health checkups. The RCS models illustrated that the odds ratio of MASLD increases with rising FLI and HSI values. FLI and HSI showed robust ability to identify MASLD (AUC: 0.90 for FLI and 0.88 for HSI). Furthermore, the sex‐stratified analysis showed that the ability of FLI and HSI to identify MASLD was significant in both sexes. A recent study evaluating Japanese participants revealed that FLI had a high AUC of 0.84 in identifying liver steatosis.^17^ Another recent Japanese study indicated that FLI and HSI are robust tools for identifying NAFLD, with AUCs of 0.88 and 0.87, respectively, which is consistent with our findings.^18^ Importantly, while previous reports examining the association between these tests and MASLD are lacking, the abovementioned studies support our results because our study showed that the population with MASLD is similar to that with NAFLD among the Japanese middle‐aged people.

Previous studies evaluated the ability of FLI and HSI to identify NAFLD using predetermined cutoff values.^13^, ^14^ Similarly, the predetermined cutoff values of FLI (30 to rule‐out and 60 to rule‐in) and HSI (30 to rule‐out and 36 to rule‐in) showed a favorable ability with high sensitivity and specificity in identifying MASLD in our study. However, there have been limited efforts to evaluate rule‐out and rule‐in cutoff values, particularly in the Asian population. In our study, we observed that the optimal, rule‐out, and rule‐in cutoff values of FLI were distinct from the predetermined cutoff values, and this distinction was particularly prominent in the sex‐stratified subgroup analyses.^13^ The drawback of FLI was also reported by a recent study, which showed that the optimal cutoff values of FLI for identifying liver steatosis differed from the original cutoff values and exhibited variations between males and females.^17^ As the FLI cutoff value was originally determined for an Italian population that has a distinct body composition compared with Asians and did not consider sex difference,^13^ its application in an Asian population requires modified cutoff values. Regarding HSI, the optimal, rule‐out, and rule‐in cutoff values in our study were close to the predetermined cutoff values. Although there were no significant differences in the AUCs of FLI and HSI, HSI may be more practical for identifying MASLD in a large‐scale Asian population setting.^14^ Notably, the cutoff value of HSI was originally derived from a Korean population and does not require waist circumference,^14^ making it more universally applicable in health checkups for all individuals.

Our study has some limitations. First, this is a single‐center study that was performed among Japanese university staff and faculty members, most of whom are desk workers. Therefore, there is a possibility of selection bias in this study, and the results may not apply to different ethnicities, professional fields, and age groups. Second, hepatic steatosis was identified by ultrasound alone without liver biopsy or other imaging techniques to evaluate steatosis and fibrosis.^39^ Third, we excluded those with moderate alcohol consumption in our study. Therefore, further multicenter studies with a larger sample size and those with moderate or excessive alcohol consumption are required to confirm the results of our study and apply indices to other types of steatotic liver disease.

In conclusion, our study evaluated the validity of the Nara declaration suggesting the use of serum ALT >30 IU/L to identify MASLD and further provided valuable evidence to validate the ability of FLI and HSI to act as indices in identifying MASLD during health checkups.

## Supporting information


**Figure S1.** Flow diagram for assessment of eligibility.
**Figure S2.** The population with steatotic liver disease among the participants (*n* = 627).
**Figure S3.** Restricted cubic spline models show the ability of the FIB‐4 index to identify MASLD.
**Table S1.** Discriminative ability of ALT to identify MASLD among obese individuals (*n* = 161).
**Table S2.** Discriminative ability of fatty liver index and hepatic steatosis index to identify MASLD among obese individuals (*n* = 161).
**Table S3.** Discriminative ability of ALT to identify MASLD among nonobese individuals (*n* = 466).
**Table S4.** Discriminative ability of fatty liver index and hepatic steatosis index to identify MASLD among nonobese individuals (*n* = 466).
