# Supplementary material for: Usefulness of health checkup‐based indices in identifying metabolic dysfunction‐associated steatotic liver disease
Source: JGH Open. 2024 Jun 17;8(6):e13110. doi: 10.1002/jgh3.13110 (PMC11183927; doi:10.1002/jgh3.13110)
Supplement: Supplementary file 1 — Figure S1. Flow diagram for assessment of eligibility. Figure S2. The population with steatotic liver disease among the participants (n = 627). Figure S3. Restricted cubic spline models show the ability of the FIB‐4 index to identify MASLD. Table S1. Discriminative ability of ALT to identify MASLD among obese individuals (n = 161). Table S2. Discriminative ability of fatty liver index and hepatic steatosis index to identify MASLD among obese individuals (n = 161). Table S3. Discriminative ability of ALT to identify MASLD among nonobese individuals (n = 466). Table S4. Discriminative ability of fatty liver index and hepatic steatosis index to identify MASLD among nonobese individuals (n = 466). [file JGH3-8-e13110-s001.docx]

**Supplementary Information**

**Usefulness of health checkup-based indices in identifying metabolic dysfunction-associated steatotic liver disease**

Takao Miwa^1, 2*^, Satoko Tajirika^1, 2^, Nanako Imamura^1^, Miho Adachi^1^, Ryo Horita^1^, Tatsunori Hanai^2^, Cheng Han Ng^3^, Mohammad Shadab Siddiqui^4^, Taku Fukao^1^, Masahito Shimizu^2^, Mayumi Yamamoto^1, 5^

^1^ Health Administration Center, Gifu University, 1-1 Yanagido, Gifu 501-1193, Japan

^2^ Department of Gastroenterology/ Internal Medicine, Graduate School of Medicine, Gifu University, 1-1 Yanagido, Gifu 501-1194, Japan

^3^ Division of Gastroenterology and Hepatology, Department of Medicine, National University Hospital, 1E Kent Ridge Road, Singapore 119228, Singapore

^4^ Division of Gastroenterology, Hepatology and Nutrition, Department of Internal Medicine, Virginia Commonwealth University, 907 Floyd Avenue, Richmond, VA 23284, USA.

^5^ United Graduate School of Drug Discovery and Medical Information Sciences, Gifu University, Gifu, 1-1 Yanagido, Gifu 501-1194, Japan

*Corresponding author: Takao Miwa, MD

Health Administration Center, Gifu University, 1-1 Yanagido, Gifu 501-1194, Japan

Tel: +81-58-233-2174

Fax: +81-58-293-2177

E-mail: miwa.takao.a6@f.gifu-u.ac.jp


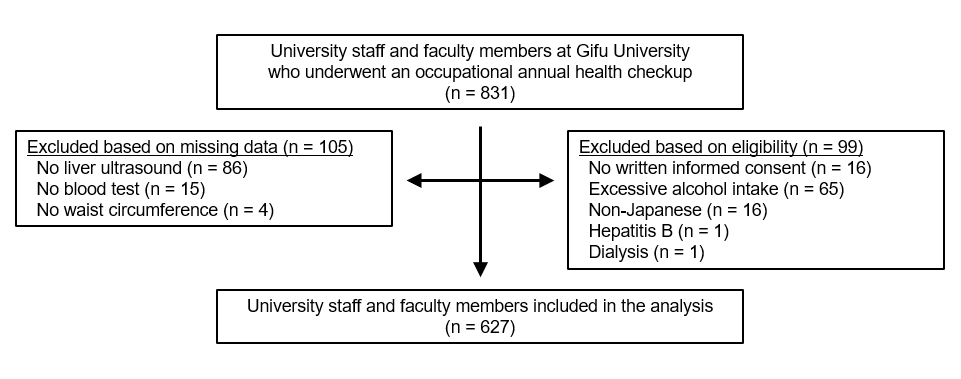
 **Figure S1.** Flow diagram for assessment of eligibility


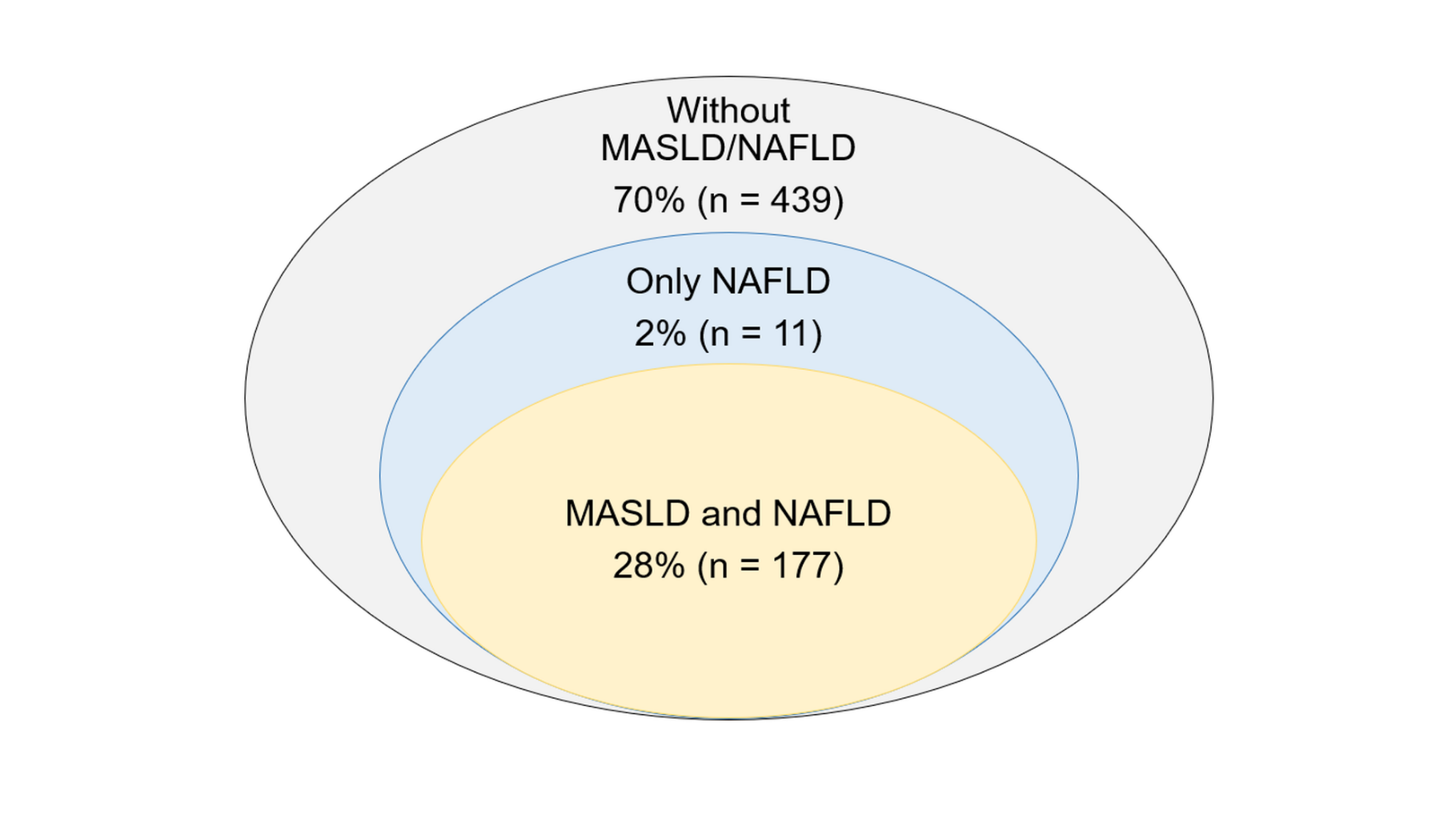
**Figure S2.** The population with steatotic liver disease among the participants (n = 627). Abbreviations: MASLD, metabolic dysfunction-associated steatotic liver disease; NAFLD, non-alcoholic fatty liver disease


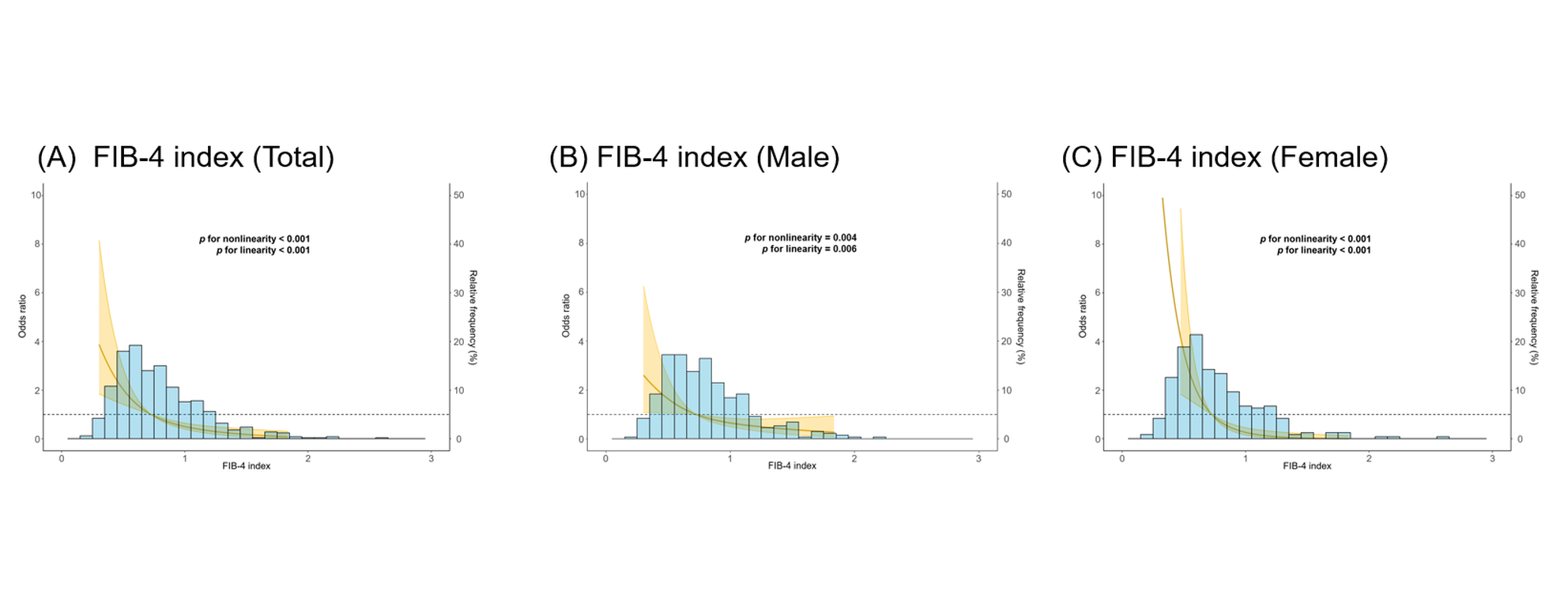
**Figure S3.** Restricted cubic spline models show the ability of the FIB-4 index to identify MASLD in (A) total cohort, (B) males, and (C) females. Abbreviation: FIB-4, fibrosis-4; MASLD, metabolic dysfunction-associated steatotic liver disease

**Table S1.** Discriminative ability of ALT to identify MASLD among obese individuals (n = 161)

|  | **ALT** | | | | |
| --- | --- | --- | --- | --- | --- |
|  | **Cutoff value** | **Sensitivity** | **Specificity** | **PPV** | **NPV** |
| **Total cohort** |  |  |  |  |  |
| Optimal | 33 | 55 | 88 | 91 | 47 |
| Rule-out | 15 | 91 | 25 | 73 | 57 |
| Rule-in | 36 | 47 | 90 | 91 | 44 |
| Predetermined | 30 | 59 | 80 | 87 | 48 |
| **Male** |  |  |  |  |  |
| Optimal | 33 | 65 | 83 | 90 | 50 |
| Rule-out | 19 | 90 | 26 | 74 | 53 |
| Rule-in | 92 | 10 | 100 | 100 | 32 |
| Predetermined | 30 | 70 | 71 | 85 | 50 |
| **Female** |  |  |  |  |  |
| Optimal | 26 | 46 | 94 | 93 | 50 |
| Rule-out | 12 | 96 | 19 | 68 | 75 |
| Rule-in | 26 | 46 | 94 | 93 | 50 |
| Predetermined | 30 | 29 | 100 | 100 | 44 |

Values are presented as percentage.

The discriminative ability of ALT was assessed at the optimal cutoff, rule-out cut off (sensitivity ≥ 90%), and rule-in cutoff (specificity ≥ 90%), and predetermined cutoff of 30.

Abbreviations: ALT, alanine amino transferase; MASLD, metabolic dysfunction-associated steatotic liver disease; NPV, negative predictive value; PPV, positive predictive value

**Table S2.** Discriminative ability of fatty liver index and hepatic steatosis index to identify MASLD among obese individuals (n = 161)

|  | **Fatty liver index** | | | | |  | **Hepatic steatosis index** | | | | |
| --- | --- | --- | --- | --- | --- | --- | --- | --- | --- | --- | --- |
|  | **Cutoff value** | **Sensitivity** | **Specificity** | **PPV** | **NPV** |  | **Cutoff value** | **Sensitivity** | **Specificity** | **PPV** | **NPV** |
| **Total cohort** |  |  |  |  |  |  |  |  |  |  |  |
| Optimal | 61 | 63 | 82 | 89 | 51 |  | 38 | 70 | 76 | 87 | 54 |
| Rule-out | 34 | 90 | 37 | 76 | 63 |  | 34 | 90 | 33 | 74 | 61 |
| Rule-in | 78 | 63 | 90 | 89 | 40 |  | 41 | 51 | 92 | 93 | 47 |
| Predetermined | 30 | 91 | 29 | 74 | 60 |  | 30 | 100 | 2 | 69 | 100 |
| Predetermined | 60 | 65 | 82 | 89 | 48 |  | 36 | 78 | 63 | 82 | 57 |
| **Male** |  |  |  |  |  |  |  |  |  |  |  |
| Optimal | 61 | 68 | 74 | 86 | 50 |  | 42 | 50 | 97 | 98 | 45 |
| Rule-out | 39 | 93 | 29 | 75 | 63 |  | 34 | 94 | 26 | 75 | 64 |
| Rule-in | 82 | 33 | 91 | 90 | 37 |  | 42 | 50 | 97 | 98 | 45 |
| Predetermined | 30 | 96 | 17 | 73 | 67 |  | 30 | 100 | 0 | 70 | NA |
| Predetermined | 60 | 68 | 74 | 86 | 50 |  | 36 | 83 | 54 | 81 | 58 |
| **Female** |  |  |  |  |  |  |  |  |  |  |  |
| Optimal | 54 | 50 | 100 | 100 | 53 |  | 38 | 57 | 94 | 94 | 56 |
| Rule-out | 20 | 93 | 50 | 78 | 80 |  | 31 | 93 | 6 | 63 | 33 |
| Rule-in | 54 | 50 | 100 | 100 | 53 |  | 38 | 57 | 94 | 94 | 56 |
| Predetermined | 30 | 75 | 56 | 75 | 56 |  | 30 | 100 | 6 | 65 | 100 |
| Predetermined | 60 | 46 | 100 | 100 | 52 |  | 36 | 64 | 81 | 86 | 57 |

Values are presented as percentage.

The discriminative ability of fatty liver index was assessed at the optimal cutoff, rule-out cut off (sensitivity ≥ 90%), rule-in cutoff (specificity ≥ 90%), and predetermined cutoff of 30 and 60.

The discriminative ability of hepatic steatosis index was assessed at the optimal cutoff, rule-out cut off (sensitivity ≥ 90%), rule-in cutoff (specificity ≥ 90%), and predetermined cutoff of 30 and 36.

Abbreviations: MASLD, metabolic dysfunction-associated steatotic liver disease; NA, not available; NPV, negative predictive value; PPV, positive predictive value

**Table S3.** Discriminative ability of ALT to identify MASLD among nonobese individuals (n = 466)

|  | **ALT** | | | | |
| --- | --- | --- | --- | --- | --- |
|  | **Cutoff value** | **Sensitivity** | **Specificity** | **PPV** | **NPV** |
| **Total cohort** |  |  |  |  |  |
| Optimal | 20 | 64 | 76 | 31 | 93 |
| Rule-out | 13 | 29 | 91 | 18 | 95 |
| Rule-in | 42 | 9 | 99 | 55 | 87 |
| Predetermined | 30 | 25 | 93 | 37 | 88 |
| **Male** |  |  |  |  |  |
| Optimal | 20 | 83 | 64 | 83 | 64 |
| Rule-out | 17 | 90 | 42 | 28 | 95 |
| Rule-in | 32 | 33 | 92 | 52 | 85 |
| Predetermined | 30 | 36 | 88 | 42 | 85 |
| **Female** |  |  |  |  |  |
| Optimal | 23 | 32 | 92 | 30 | 93 |
| Rule-out | 10 | 92 | 15 | 11 | 95 |
| Rule-in | 22 | 32 | 90 | 26 | 92 |
| Predetermined | 30 | 8 | 97 | 20 | 91 |

Values are presented as percentage.

The discriminative ability of ALT was assessed at the optimal cutoff, rule-out cut off (sensitivity ≥ 90%), and rule-in cutoff (specificity ≥ 90%), and predetermined cutoff of 30.

Abbreviations: ALT, alanine amino transferase; MASLD, metabolic dysfunction-associated steatotic liver disease; NPV, negative predictive value; PPV, positive predictive value

**Table S4.** Discriminative ability of fatty liver index and hepatic steatosis index to identify MASLD among nonobese individuals (n = 466)

|  | **Fatty liver index** | | | | |  | **Hepatic steatosis index** | | | | |
| --- | --- | --- | --- | --- | --- | --- | --- | --- | --- | --- | --- |
|  | **Cutoff value** | **Sensitivity** | **Specificity** | **PPV** | **NPV** |  | **Cutoff value** | **Sensitivity** | **Specificity** | **PPV** | **NPV** |
| **Total cohort** |  |  |  |  |  |  |  |  |  |  |  |
| Optimal | 14 | 81 | 79 | 39 | 96 |  | 31 | 72 | 81 | 39 | 94 |
| Rule-out | 8 | 91 | 62 | 29 | 98 |  | 28 | 90 | 54 | 25 | 97 |
| Rule-in | 25 | 49 | 90 | 45 | 91 |  | 33 | 52 | 90 | 48 | 92 |
| Predetermined | 30 | 43 | 92 | 48 | 91 |  | 30 | 79 | 72 | 33 | 95 |
| Predetermined | 60 | 7 | 99 | 63 | 87 |  | 36 | 21 | 97 | 58 | 88 |
| **Male** |  |  |  |  |  |  |  |  |  |  |  |
| Optimal | 16 | 88 | 61 | 36 | 95 |  | 33 | 69 | 80 | 69 | 81 |
| Rule-out | 15 | 90 | 60 | 36 | 96 |  | 28 | 93 | 34 | 26 | 95 |
| Rule-in | 36 | 48 | 90 | 54 | 87 |  | 35 | 40 | 91 | 53 | 86 |
| Predetermined | 30 | 57 | 83 | 46 | 89 |  | 30 | 86 | 52 | 31 | 94 |
| Predetermined | 60 | 10 | 98 | 57 | 81 |  | 36 | 31 | 94 | 57 | 85 |
| **Female** |  |  |  |  |  |  |  |  |  |  |  |
| Optimal | 11 | 76 | 89 | 76 | 90 |  | 29 | 80 | 78 | 29 | 97 |
| Rule-out | 6 | 96 | 69 | 25 | 99 |  | 26 | 96 | 36 | 14 | 99 |
| Rule-in | 12 | 72 | 91 | 47 | 97 |  | 31 | 48 | 94 | 48 | 94 |
| Predetermined | 30 | 20 | 99 | 63 | 92 |  | 30 | 72 | 87 | 37 | 97 |
| Predetermined | 60 | 4 | 100 | 100 | 91 |  | 36 | 4 | 100 | 100 | 91 |

Values are presented as percentage.

The discriminative ability of fatty liver index was assessed at the optimal cutoff, rule-out cut off (sensitivity ≥ 90%), rule-in cutoff (specificity ≥ 90%), and predetermined cutoff of 30 and 60.

The discriminative ability of hepatic steatosis index was assessed at the optimal cutoff, rule-out cut off (sensitivity ≥ 90%), rule-in cutoff (specificity ≥ 90%), and predetermined cutoff of 30 and 36.

Abbreviations: MASLD, metabolic dysfunction-associated steatotic liver disease; NPV, negative predictive value; PPV, positive predictive value
